# Evaluating outcomes of a three-year case management program for mothers with prenatal substance use according to race/ethnicity, Washington State, 2006–2017

**DOI:** 10.1186/s12889-023-16670-z

**Published:** 2023-09-20

**Authors:** Susan A. Stoner, J. Christopher Graham, Therese M. Grant

**Affiliations:** Addictions, Drug & Alcohol Institute, Department of Psychiatry & Behavioral Sciences, University of Washington School of Medicine, 1107 NE 45th St., Suite 120, Box 354805, Seattle, WA 98195-4805 USA

## Abstract

**Background:**

Well-designed public health interventions ideally aspire to reduce health disparities between racial and ethnic groups. Yet, there remains virtually no research examining racial/ethnic disparities in interventions for marginalized perinatal populations with substance use disorders (SUD). We sought to examine whether there were racial/ethnic differences at intake, in retention, and in program outcomes among pregnant or postpartum women with prenatal substance use enrolled in a three-year intensive case management intervention. We hypothesized that: (1) at baseline, numerous racial/ethnic disparities in well-being, health, and health care would be observed, and (2) after the three-year intervention few racial/ethnic disparities in maternal and child health and welfare would be found.

**Methods:**

We used self-reported data from 3,165 women aged 18 to 45 years enrolled in the Parent-Child Assistance Program in Washington State between May 10, 2006, and September 21, 2017. We used Fisher-Freeman-Halton Exact Tests and *t*-tests to compare racial/ethnic groups at program enrollment and exit and logistic regression to examine likelihood of completing the intervention by group, controlling for other factors.

**Results:**

Despite numerous racial/ethnic differences at enrollment, there were no such differences in outcomes among those who finished the program and completed an exit interview. Different racial/ethnic groups received comparable case manager time. American Indians/Alaska Natives were less likely to finish the program (Adjusted Odds = 0.66).

**Conclusions:**

Participants who finished the program achieved comparable outcomes regardless of race/ethnicity. More work is needed to understand why American Indian/Alaska Native women were less likely than the others to finish the program and to close this service gap.

## Introduction

People from minoritized racial/ethnic groups continue to experience disparities in well-being, health, and health care compared to White counterparts, particularly with regard to maternal and child health and welfare [1–3] and access to behavioral health treatment, [4] including mental health services [5] and substance use disorder (SUD) treatment [6]. To address such persistent and pernicious disparities, thought leaders have called for comprehensive approaches to quality improvement, including developing new models of care for pregnant and postpartum mothers, [1] enhancing services to address social determinants of health, [7] tailoring provision of care to reduce obstacles to behavioral health care, [4] responding to patients’ needs and preferences, [4] addressing perceived treatment need, [8] and expanding the use of patient navigation and case management [9].

The Parent-Child Assistance Program (PCAP) is a three-year case management intervention for pregnant and postpartum women with problematic substance use during their most recent pregnancy, that is, substance use that creates problems in the women’s lives or puts their children at risk of harm either prenatally or postnatally. Grounded in relational theory, [10] subscribing to the transtheoretical model of behavior change, [11] and embracing principles of harm reduction, [12] PCAP provides each participant with a highly-trained bachelor’s-level case manager, whose service standard is to meet with the participant twice per month in community settings, visiting them at home whenever possible. Case managers coach participants in setting their own goals and taking steps to achieve them, provide practical assistance (e.g., occasional transportation), serve as role models, and offer emotional support and encouragement. Case managers connect participants to needed community resources and recovery supports, including but not limited to housing, health care, SUD treatment, mental health treatment, legal aid, job training, and educational development, with an array tailored to the participant’s needs and goals. On average, case managers carry a caseload of 16 participants, receive at least biweekly individual clinical supervision by a master’s-level clinician, biweekly group supervision, and individual consultation with their supervisor whenever needed. Case managers work with the dyad (participant and the index child) and other family members in support of the participant’s goals and recovery.

Developed in 1991, PCAP has long-standing evidence of efficacy [13–16]. For example, past analyses have shown that only 12% of participants had a subsequent substance-exposed birth during the program [17]. By contrast, 21% of similar mothers over a comparable time period who received substance use disorder treatment without case management had a subsequent substance-exposed infant [17]. Given the long-term, comprehensive, intensive, and tailored nature of the PCAP intervention, we sought to examine whether PCAP avoids racial/ethnic disparities in outcomes in a diverse cohort of pregnant and parenting women, reasoning that effectively addressing each mother’s particular needs would help to do so. We hypothesized that: (1) at baseline, numerous racial/ethnic disparities in well-being, health, and health care would be observed, and (2) there would be no racial/ethnic disparities in outcomes of the three-year intervention.

To date, there is virtually no research examining racial/ethnic disparities in outcomes of case management programs or interventions for marginalized perinatal populations with SUD. While research increasingly reports race and ethnicity of persons served, data on program outcomes moderated by race or ethnicity usually are not presented, obscuring important information about potential inequities in program effectiveness. This study contributes to the public health literature by examining whether a long-term, personalized intervention can help to close racial/ethnic gaps in a population of women with complex risk profiles.

## Method

The Washington State Institutional Review Board fully reviewed and approved (D-030310) the study protocol. All participants provided informed, signed consent for their data to be used for research purposes. All methods were carried out in accordance with relevant guidelines and regulations.

### Participants

**Fig. 1:**
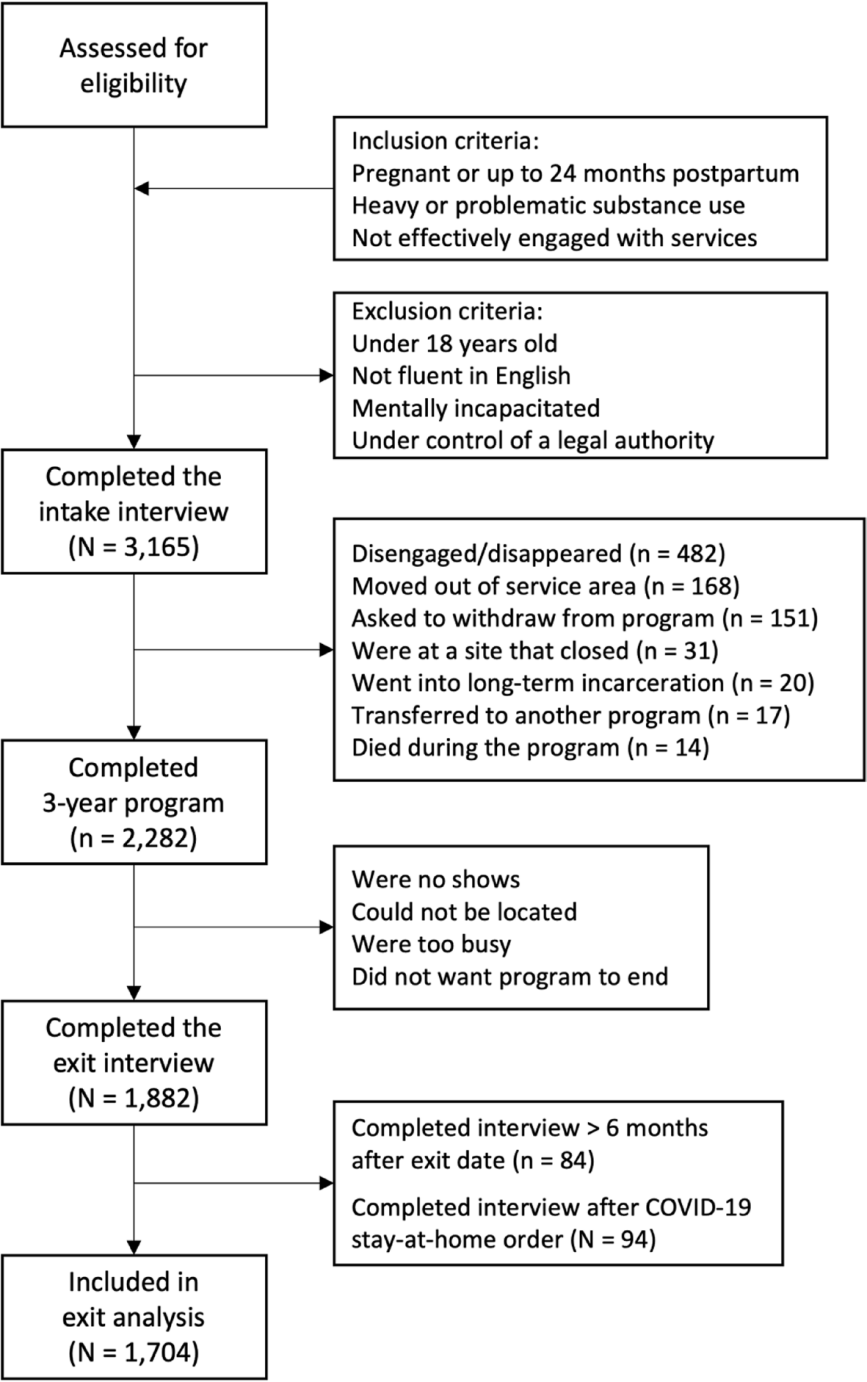
Inclusion and exclusion criteria and flow of participants through the program and study

We examined data for all consenting participants who enrolled in PCAP in Washington State between May 10, 2006, and September 21, 2017. The cutoff point was selected to exclude participants whose three-year exit data could have been affected by the COVID-19 pandemic. During the chosen timeframe, a total of 3,165 eligible women were enrolled at twelve program sites serving fifteen Washington counties, completed an intake interview in which they reported their race and ethnicity and consented for their data to be used for research. Inclusion and exclusion criteria are shown in Fig. 1. Most participants were referred by community providers (e.g., child welfare workers, SUD treatment providers) who were familiar with the intervention (through brochures, presentations, and word of mouth).

### Analysis sample

Figure 1 shows the flow of participants through the program and the study. Of the 3,165 participants, 883 (27.9%) did not complete the program because they disengaged or disappeared (*n* = 482), moved out of the area (*n* = 168), withdrew from the program (*n* = 151), died (*n* = 14), went into long-term incarceration (*n* = 20), transferred to another program (*n* = 17), or were at a site that closed (*n* = 31). An additional 400 mothers (12.6%) were enrolled in PCAP for three years but did not provide data for an exit interview (were no-shows, could not be located, were too busy, or did not want to end PCAP). Data for 178 women (5.6%) were excluded from the analysis of the exit data because, though they exited the program, they completed their exit interview either more than six months after their target exit date (*n* = 84) or after the governor’s Stay-at-Home order to address the COVID-19 pandemic on March 23, 2020 (*n* = 94). Data from the remaining 1,704 were included in the analysis of the outcome data.

#### Interviewers

Intake interviewers were PCAP clinical supervisors, all of whom had extensive experience working with diverse women. Exit interviewers were trained research assistants who did not have contact with participants during the intervention. All interviewers used detailed instruction manuals (available upon request) and were trained by an expert in the Addiction Severity Index to standards designed to ensure consistent interview procedures and enhance the reliability and validity of the data.

### Measures

#### Demographics

The following demographics were assessed as part of the Addiction Severity Index [18] interview, described in further detail below: age (number of years, calculated from date of birth), race and ethnicity (select all that apply), marital status, employment status, housing status, education (number of years), and household income (dollars per month). We also recorded whether they were enrolled at site in a rural or urban county according to state guidelines [19].

#### Racial/ethnic categorization

Our analysis was based upon the reporting standard of the Washington State Department of Children, Youth and Families (DCYF) for advancing racial equity [20]. The WSRDAC/M Reporting Standard combines racial and ethnic (Hispanic/Latino) identifications into a single race/ethnicity categorical variable. Reflecting the degree of disparities generally observed in the child welfare system, individuals with more than one racial/ethnic identifier are hierarchically assigned (depending on the particulars) to the American Indian/Alaska Native (abbreviated AI/AN), African American/Black (abbreviated Black), or Hispanic/Latino (abbreviated Hispanic) “multiracial” WSRDAC/M categories. Procedurally, participants with any identification as American Indian/Alaska Native are categorized as such; then any remaining participants with any identification as Black/African American are categorized as such; and then any remaining participants with any identification as Hispanic/Latino are categorized as such. Finally, any remaining multiracial participants would have identified as Asian/Pacific Islander and are categorized as such. As a result, only the White category does not include any multiracial participants [20]. For analysis purposes, rather than use a polytomous race/ethnicity variable, we created four dichotomous variables that reflected membership in each of the minoritized groups.

#### Addiction severity index

At program intake and exit, we interviewed participants with the Addiction Severity Index (ASI), 5th edition, [18, 21] a widely used standardized, semi-structured interview nstrument with well-supported reliability and validity [22]. It assesses medical, employment/support, alcohol, drug, legal, family/social, and psychiatric domains. These domains are of interest because they may have a significant impact on participants’ ability to benefit from the program and may differ by race and ethnicity at program intake. We created a dichotomous indicator of illicit drug use within the past 30 days at exit that excluded use of alcohol, cannabis, and prescription drugs used as prescribed.

#### Supplemental questions for pregnant and postpartum women

We developed supplemental items, worded by subject matter experts in a straightforward manner, to assess drug use during pregnancy, reproductive history, and adverse life experiences not assessed in the ASI and administered them in conjunction with the intake ASI. The substances assessed were the same as those in the standard ASI: alcohol, heroin, methadone, other opiates/analgesics, barbiturates, other sedatives/tranquilizers, cocaine, amphetamines, cannabis, hallucinogens, and inhalants. We created a dichotomous variable for each substance to indicate whether it was used during pregnancy (or the month prior) and an indicator of illicit drug use that excluded use of alcohol, cannabis, and prescription drugs. We included an indicator of binge alcohol use, defined as ≥ 4 standard drinks per occasion [23]. Questions about reproductive history assessed birth control use (no use, sporadic use, or regular use), the number of the participant’s biological children and, of those, the number not living with the participant. Questions about adverse life experiences asked whether the participant’s natural mother drank alcohol while she was pregnant with the participant and whether child protective services were involved when the participant was growing up.

#### Recovery supports/community services

At PCAP intake and at exit, participants were asked to identify services they had received in the preceding year. Services assessed included those related to healthcare provision for mother and child(ren), family planning, mental health, housing, emergency funds, food/clothing/supplies, legal aid, domestic violence, childcare, support groups, public schools, and public health nursing. Each service was coded as to whether the client herself expressed a need for that service (Yes = 1/No = 0) and whether the service was received (Yes = 1/No = 0). For each participant, a service ratio was calculated as the sum of the number of services reported as received divided by the sum of the number reported as needed. A ratio of one indicates that all needs were met, including any who reported no needs and thus had no unmet needs.

#### Case manager time spent

On a weekly basis (i.e., a seven-day period), case managers recorded the amount of time spent working directly with or on behalf of each participant. We calculated a summary score for direct time by summing the number of contact hours with the CM during the intervention (including face-to-face meetings, time spent transporting the participant, and phone communication with the participant), as well as a summary score for “other” time spent working on a participant’s case (i.e., without the participant present), which is the sum of time spent working with an agency on behalf of the participant, time spent with participant’s family, and time spent corresponding with the participant.

### Data analytic approach

All descriptive analyses were based solely on the responses provided. We generated intake descriptive bivariate results using IBM® SPSS® Statistics version 28.0, examining family composition, maternal characteristics (demographic and psychosocial), and community services (needed) variables by participant race/ethnicity (as defined above). With one exception, to examine nominal variables we used the Fisher-Freeman-Halton Exact Test to examine nominal variables. The exception (necessary for computational reasons) was the urban/rural site indicator, for which we referred to the Pearson Chi-Square test result. Post-hoc, we used cell-specific adjusted standardized residuals to gauge each individual cell’s contribution to the statistically significant result. For testing differences of the means of continuous variables across the race/ethnicity groups we entered (as a fixed set) four race/ethnicity indicators (using White as the reference category) into a regression equation and determined statistical significance of each race/ethnicity indicator by reference to the *t*-tests of their regression coefficients. As these tests were for descriptive purposes, rather than hypothesis testing, we made no correction for multiple tests, and all tests were two tailed (with *p* < .05).

In considering possible limits to the study’s generalizability, we conducted bivariate tests for the same set of family/maternal/service variables, again using the Fisher-Freeman-Halton Exact Test, but for this analysis we examined them by a categorical variable including three study groups: (1) Attrition Group (*N* = 852), (2) Exited with No Interview Group (*N* = 400), and (3) Outcome Group (*N* = 1,704). We did not include in this analysis participants whose site closed while they were in the program (*N* = 31) or whose exit interview was more than six months beyond their target interview date (*N* = 178).

We conducted an attrition analysis for two reasons: to consider whether exit outcomes may have been biased by who remained in the study and to examine whether there were racial/ethnic differences in intervention completion. As the first part of the attrition analysis, primarily with the purpose of screening variables into a logistic regression that was to follow (given the large number of variables involved), we conducted the same procedure as in the preceding paragraph but included only the Attrition and Outcome groups (*N* = 2,556). The second part of the attrition analysis used binary logistic regression with listwise deletion, including as candidate predictors the variables screened in by the prior step, to determine (now in a multivariable context) the variables significantly associated with Outcome Group membership as the outcome. We used the Backward Entry Likelihood Ratio (LR) Method and tested to see whether the resulting final model was the same if we used the Forward Entry LR Method instead.

For participants with exit data, we used Fisher-Freeman-Halton Exact Tests and *t*-tests of regression coefficients to examine bivariate associations of participant race/ethnicity with PCAP case manager time spent on case, participant’s child welfare/criminal justice involvement, SUD treatment received (at any time during PCAP), and other services needed and received (both during the final year of PCAP), as well as the ratio of services received to services needed. Finally, using the same methods, we examined participant outcomes by participant race/ethnicity.

## Results

Below and in Table 1 we summarize self-reported maternal demographics, history, mental health, and prenatal substance use and service needs at program intake by race/ethnicity.

**Table 1:** Maternal demographics, history, psychiatric and prenatal substance use characteristics, and service needs self-reported at intake, among each racial/ethnic group (*N* = 3,165). Dichotomous variables are expressed as percentages while continuous variables are expressed as means (SDs)

	Total N in Analysis	American Indian/ Alaska Native *N* = 669	Asian/ Pacific Islander *N* = 63	African American/ Black *N* = 257	Hispanic/ Latino *N* = 306	White *N* = 1,870^a^	Overall % or Mean (SD)^b^
Rural Site	3,165	45.7%	38.1%	**27.6% ↓**	**64.1% ↑**	49.4%	48.0%^***c^
**Maternal social history**							
Education (years completed)	3,163	↓11.0 (1.7)^***^	11.2 (1.5)	11.2 (1.8)	↓10.7 (2.0)^***^	11.3 (1.7)	11.2 (1.7)
Usually employed in past 3 years	3,146	22.6%	**37.7% ↑**	25.5%	20.3%	22.0%	22.5%^*^
Number of children^d^ (not including index child)	3,160	↑1.8 (1.7)^***^	1.3 (1.6)	**↑1.7**^**⁎**^**(1.6)**	↑2.0 (1.6)^***^	1.4 (1.4)	1.6 (1.5)
Number of biologic children living with participant	3,160	0.4 (0.9)	0.4 (0.8)	0.4 (0.9)	↑0.6 (1.1)^***^	0.3 (0.7)	0.4 (0.8)
Child welfare involved (during own childhood)	3,152	**36.2% ↑**	19.4%	26.2%	**17.7% ↓**	**21.1% ↓**	24.4%^***^
One or both parents had alcohol/drug problems	3,144	**93.4% ↑**	**68.3% ↓**	85.9%	84.5%	86.7%	87.4%^***^
Own mother drank while pregnant with participant	2,455	**23.0% ↑**	**1.9% ↓**	12.4%	**9.3% ↓**	**11.9% ↓**	13.8%^***^
Ever incarcerated (since age 18)^e^	3,148	**81.2% ↑**	66.7%	80.0%	76.4%	**72.4% ↓**	75.1%^***^
How many times incarcerated (since age 18)?	3,148	↑8.2 (14.0)^***^	4.9 (8.8)	↑8.5 (16.4)^***^	5.7 (11.4)	5.5 (10.1)	6.3 (11.8)
**Lifetime psychiatric symptoms**							
Depression	3,155	75.7%	79.4%	80.5%	**69.6% ↓**	77.0%	76.3%^*^
Anxiety	3,156	**72.0% ↓**	77.8%	**82.4% ↑**	**64.8% ↓**	78.2%	76.0%^***^
Hallucinations	3,156	13.5%	6.3%	**19.1% ↑**	13.2%	12.0%	12.9%^*^
Violent behavior	3,155	45.2%	38.1%	**50.2% ↑**	42.6%	**40.5% ↓**	42.4%^*^
Suicidal thoughts	3,154	38.1%	46.0%	**47.3% ↑**	**35.3% ↓**	42.1%	41.1%^*^
**Substance use during pregnancy**							
Any alcohol	2,986	**47.8% ↑**	41.3%	**49.2% ↑**	39.9%	**37.8% ↓**	41.1%^***^
Binge alcohol (≥ 4 drinks per occasion)	2,957	**32.5% ↑**	22.2%	29.8%	22.9%	**22.2% ↓**	25.1%^***^
Heroin	2,929	28.5%	26.2%	**21.9% ↓**	**24.5% ↓**	**33.4% ↑**	30.4%^***^
Other illicit opiates	2,934	**38.2% ↑**	39.3%	29.8%	**26.5% ↓**	35.3%	34.7%^**^
Cocaine	2,910	**20.3% ↑**	12.7%	**32.1% ↑**	15.4%	**12.5% ↓**	16.0%^***^
Methamphetamine	3,000	**51.9% ↓**	49.2%	**47.5% ↓**	**68.4% ↑**	**61.6% ↑**	58.8%^***^
Cannabis	2,991	49.8%	52.4%	58.0%	**43.8% ↓**	**54.9% ↑**	53.0%^**^
Nicotine	3,017	81.2%	77.8%	80.9%	**67.4% ↓**	**85.1% ↑**	82.1%
**Self-reported service needs at intake**							
Mental health care (participant)	3,154	72.6%	77.8%	79.3%	**65.1% ↓**	**77.3% ↑**	75.3%^***^
Public housing	3,153	72.6%	73.0%	**82.4% ↑**	**62.5% ↓**	**75.6% ↑**	74.2%^***^
Emergency housing	3,153	39.5%	31.7%	**46.9% ↑**	**31.3% ↓**	41.5%	40.3%^**^

^a^For comparisons of means the reference group is *White*. For tests of association there is no comparison group (expected values are based upon the marginals).

^b^Fisher-Freeman-Halton Exact Test (categorical variables) or t-test of individual regression coefficients (continuous variables).

^c^Based upon two-sided Chi-Square test.

^d^Number of children participant reported she had at PCAP baseline, (whether or not they were living with her) minus 1 (to exclude index child).

^e^Since age 18; includes # times the participant has been jailed and charged (whether or not the charge resulted in a conviction).

↑↓ (1) *Means* of groups that are significantly different from those of the white participants (judging by regression results) and (2) *Percentages* (as indicated by adjusted standardized residuals, treated as post hoc tests) that are significantly different from expected values (judging by two-sided exact tests) are in bold font marked by a ↑ symbol (higher value) or a ↓ symbol (lower value) ^⁎^*p <* .05; ^⁎⁎^*p <* .01; ^⁎⁎⁎^*p <* .001

### Baseline differences between groups

#### Maternal demographics

We found no statistically significant differences at PCAP enrollment across race/ethnicity with respect to maternal age at enrollment (overall mean = 26.7 years, SD = 5.2), proportion married (9.1%), living in stable housing (28.5%), receiving any welfare (60.3%), living below the federal poverty level (93.0%), and index child living with their mother/PCAP participant (69.4%). As shown in Table 1, nearly half of the participants were enrolled at rural intervention sites, with significantly lower proportions of Black women and higher proportions of Hispanic women in the rural areas. Compared to White women, AI/AN and Hispanic women reported significantly fewer mean years of education. A significantly higher proportion of Asian/PI women had been employed in the past three years. Compared to White women, AI/AN, Hispanic, and Black women had significantly more children (not including the index child), and Hispanic women had more of their children living with them.

#### Maternal history

We found no statistically significant differences by race/ethnicity in lifetime physical abuse (74.3% overall) or sexual abuse (58.0% overall) or whether participants had been hit by a sexual partner as an adult (73.6% overall). As shown in Table 1, AI/AN women (as children) were significantly more likely than would be expected to have had child welfare involvement with their family, parent(s) with alcohol or drug problems, heavy prenatal alcohol exposure, and adult incarceration.

#### Maternal mental health

In response to ASI questions regarding lifetime psychiatric symptoms, Black women, as shown in Table 1, reported significantly higher than expected rates of anxiety, hallucinations, violent behavior, and suicidal thoughts. Hispanic women reported lower than expected rates of depression, anxiety, and suicidal thoughts. AI/AN women reported lower than expected rates of anxiety.

#### Substance use during pregnancy

Patterns of substance use varied by race/ethnicity (Table 1). AI/AN and Black participants reported significantly higher rates than expected of alcohol and cocaine use; AI/AN women had significantly higher rates of binge alcohol and illicit methadone use. White women reported significantly higher proportions than expected of heroin, cannabis, and nicotine use. Hispanic and White women reported the highest rates of illicit amphetamine use.

#### Community service needs at intake

There were few racial differences in service needs expressed at intake. Similar proportions of participants across racial groups expressed needs for family planning (overall 85.3%), SUD recovery support group (85.0%), child healthcare provider (67.0%), legal aid (52.8%), domestic violence support (34.6%), and childcare (28.6%). As shown in Table 1, White women expressed significantly higher needs for mental health care and public housing. Black women expressed significantly higher than expected needs for emergency and public housing. Hispanic women expressed lower than expected needs for these three services.

Below and in Table 2, we summarize PCAP case management time (i.e., intervention dosage) and community services involvement during the intervention by race/ethnicity among all participants with exit data.

**Table 2:** Community services needed and received (of those who expressed a need) during the final year of PCAP, by race/ethnicity among 1,704 participants with exit data. Dichotomous variables are expressed as percentages while continuous variables are expressed as means (SDs)

	Total N in Analysis	American Indian/ Alaska Native *N* = 329	Asian/ Pacific Islander *N* = 40	African American/ Black *N* = 126	Hispanic/ Latino *N* = 166	White *N* = 1,043^a^	Overall % or Mean (SD)^b^
**Childcare/daycare**							
Expressed need	1,676	**44.4% ↓**	59.0%	57.3%	**58.4% ↑**	50.8%	51.0%^*^
Service received	855	84.0%	100.0%	91.5%	81.4%	87.5%	86.9%
**Family healthcare provider**							
Expressed need	1,604	90.3%	86.8%	93.3%	92.5%	92.4%	92.0%
Service received	1,475	99.6%	100.0%	100.0%	98.6%	98.8%	99.1%
**Mental health**							
Expressed need	1,688	64.8%	68.4%	68.8%	62.4%	61.1%	62.7%
Service received	1,058	67.0%	57.7%	60.5%	**48.5% ↓**	64.0%	62.7%^*^
**Family planning, birth control**							
Expressed need	1,685	59.1%	56.4%	55.2%	**70.9% ↑**	57.1%	58.7%^*^
Service received	989	71.4%	77.3%	62.3%	72.6%	69.8%	70.1%
**Alcohol/drug support group**							
Expressed need	1,689	61.0%	64.1%	65.6%	66.7%	61.2%	62.1%
Service received	1,049	77.4%	**60.0% ↓**	79.3%	**64.5% ↓**	**81.7% ↑**	78.4%^***^
**Domestic violence support**							
Expressed need	1,686	19.0%	17.9%	24.0%	27.1%	20.9%	21.3%
Service received	359	53.2%	42.9%	53.3%	46.7%	54.4%	52.9%
**Public housing**							
Expressed need	1,683	63.8%	66.7%	77.6%	67.3%	68.1%	67.9%
Service received	1,142	55.3%	57.7%	54.6%	62.2%	51.1%	53.4%
**Emergency housing**							
Expressed need	1,688	22.9%	25.6%	27.2%	27.7%	21.0%	22.6%
Service received	381	52.0%	30.0%	38.2%	34.8%	45.4%	44.4%
**Legal services**							
Expressed need	1,687	32.8%	33.3%	**52.0% ↑**	33.1%	34.9%	35.6%^**^
Service received	600	82.2%	76.9%	72.3%	61.8%	70.6%	72.2%
**Total Number service needs** (of nine)	1,691	4.5 (1.9)	4.7 (1.8)	↑ 5.2^**^(1.7)	↑ 5.0^*^(2.0)	4.6 (1.9)	4.7 (1.9)
**Total Number services received** (of nine)	1,691	3.4 (1.6)	3.4 (1.6)	↑ 3.7^*^(1.4)	3.4 (1.6)	3.4 (1.6)	3.4 (1.6)
**Ratio of services received to services needed**	1,691	0.77 (0.25)	0.77 (0.27)	0.74 (0.22)	↓ 0.72^*^(0.28)	0.77 (0.25)	0.76 (0.25)

^a^For comparisons of means the reference group is *White*. For tests of association there is no comparison group (expected values are based upon the marginals)

^b^Fisher-Freeman-Halton Exact Test (categorical variables) or t-test of individual regression coefficients (continuous variables)

↑↓ (1) *Means* of groups that are significantly different from those of the White participants (indicated by regression results) and (2) *Percentages* (as indicated by adjusted standardized residuals, treated as post hoc tests) that are significantly different from expected values (judging by two-sided exact tests) are in bold font marked by a ↑ symbol (higher value) or a ↓ symbol (lower value)

^⁎^*p <* .05; ^⁎⁎^*p <* .01; ^⁎⁎⁎^*p <* .001

### During the program

#### Dosage of PCAP intervention

Among participants with exit data, the mean amount of direct face-to-face case manager time received was 0.98 h in a 7-day period (SD = 0.54), and the mean amount of other case manager time spent on behalf of the participant was 0.22 h in a 7-day period (SD = 0.15). Time received was comparable across racial groups though Hispanic participants received more direct time (mean = 1.11, SD = 0.63) and AI/AN participants received more other time (mean = 0.23, SD = 0.16) compared to White participants (direct time mean = 0.97, SD = 0.52, *p* < .05; other time mean = 0.21, SD = 0.15, *p* < .05).

#### Criminal justice involvement

Criminal justice involvement was of particular interest due to the extant racial and ethnic disproportionalities substantiated in the literature [24, 25]. Across racial groups, during the program, participants were similarly likely to be involved with the criminal justice system. Overall, only 1.8% were charged with major charges, including homicide, rape, arson, robbery and weapons charges; 26.9% were charged with lesser charges, including shoplifting/vandalism, drug charges, forgery, identity theft, burglary/larceny/breaking and entering, other theft, assault, prostitution, possession of stolen property, disorderly conduct, harassment or parenting offenses; and 15.2% were charged with procedural violations, such as contempt of court, parole/probation violations, failure to appear, failure to pay fines, etc. The only racial difference was in major driving violations, which were observed at higher rates than expected among Hispanic participants (36.8%, *p* < .05) and lower rates among Asian/PI participants (12.8%, *p* < .05).

#### Substance use treatment

Rates of treatment receipt during the program were comparable across racial groups. By the end of the three-year program, 56.5% of participants had completed or were still attending inpatient treatment, 82.8% of participants had completed or were still attending outpatient treatment, and 87.8% had received either form of treatment. More than three-quarters (76.3%) had a period of abstinence from drugs and alcohol for six months or more during the program.

#### Community service needs expressed and received

Table 2 shows the proportion of participants who reported at exit their need for community services during their final year in PCAP and whether they received those services. Note that, given the long duration of the program and dynamic nature of mothers’ lives, needs identified during the final year of the program and those at intake may have been quite different. In the year leading up to exit, compared to other groups, Black women were more likely to need legal services (52%) and as likely to receive them (72.3%) while Hispanic women were more likely to need childcare (58.4%) and family planning (70.9%) and as likely to receive those services (81.4% and 72.6% respectively). Hispanic women expressed similar needs for mental health services and alcohol/drug support as other racial/ethnic groups, but they were less likely to have those needs met. Asian/PI women were less likely to receive alcohol/drug support group services. Overall, Hispanic women had the lowest ratio of services received to services needed compared to White participants (0.72 vs. 0.77, *p* < .05).

### Program Impact at 3-year exit

**Fig. 2:**
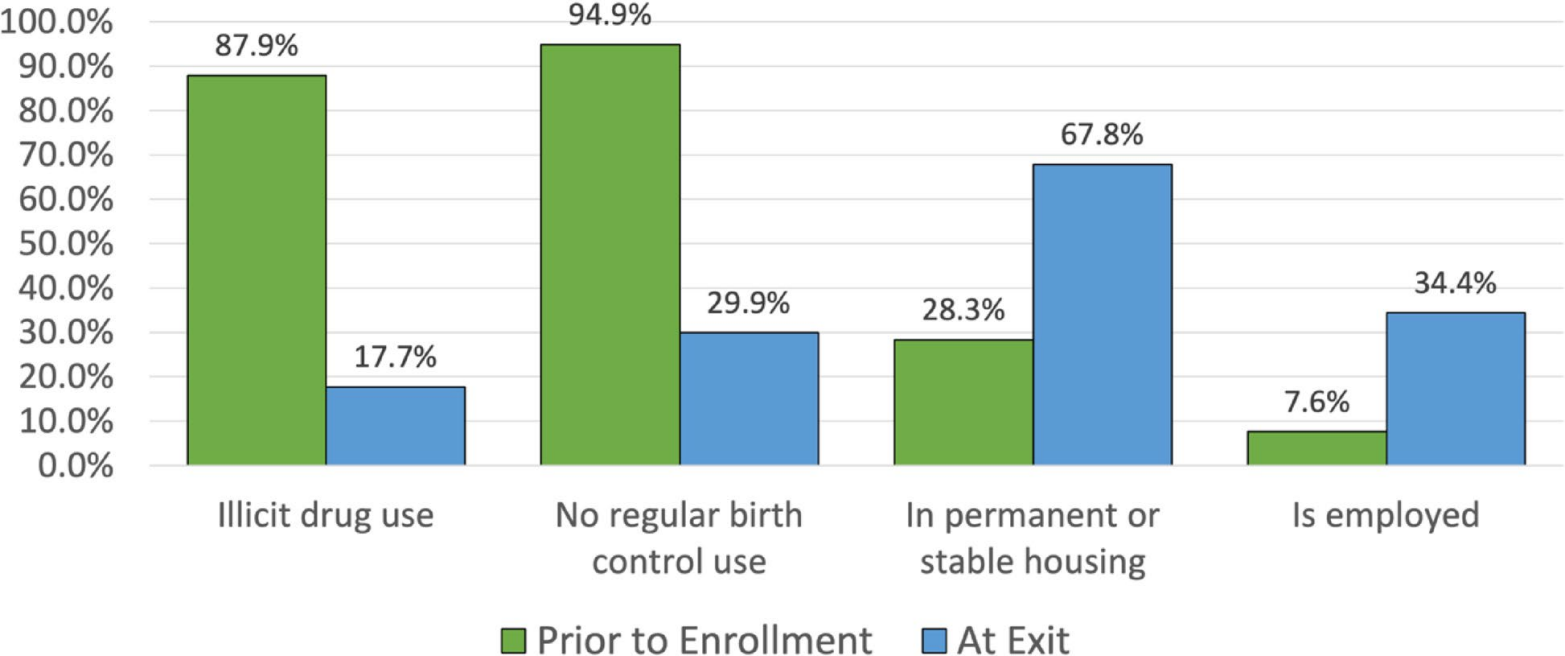
Key indicators of program benefit by participant self-report, prior to program enrollment versus at program exit

Outcomes did not differ significantly by race/ethnicity. Key outcomes are shown in Fig. 2. Across racial/ethnic groups, 87.9% reported use of illicit drugs during pregnancy, while at exit, only 17.7% had done so in the past 30 days. Around the time of becoming pregnant, 94.9% had not been using birth control regularly, while at exit, 29.9% were not doing so. At intake, 28.3% were stably housed and 7.6% were employed. At exit, those rates were 67.8% and 34.4%, respectively. Two-thirds (66.7%) had physical custody of the index child, and 50.4% had attended or completed a training or education program. These participant outcomes did not differ significantly by race/ethnicity.

#### Predictors of attrition from the program

Table 3 presents results indicating that, controlling for other variables in the model via logistic regression, completing the three-year intervention and providing exit data was negatively associated with AI/AN self-identification (Adjusted Odds Ratio, AOR = 0.66), lifetime number of times incarcerated (AOR = 0.99), and heroin use during pregnancy (AOR = 0.72). Conversely, intervention completion was positively associated with having physical custody of the index child at intake (AOR = 1.44), having a history of physical abuse (AOR = 1.31), needing childcare at intake (AOR = 1.24), and needing public housing at intake (AOR = 1.59).

**Table 3:** Factors associated with completion versus attrition from the three-year intervention program among *N* = 2,556 participants included in the analysis

	Adjusted Odds Ratio 95% CI	p
**Variables Associated with Attrition**		
American Indian/Alaska Native racial identification^a^	0.66 (0.54, 0.82)	< 0.001
Incarceration – lifetime number of times	0.99 (0.98, 1.00)	0.005
Heroin use during pregnancy^a^	0.72 (0.59, 0.88)	0.001
**Variables Associated with Complettion**		
Physical custody of index child at intake^a^	1.44 (1.19, 1.75)	< 0.001
Physical abuse – any lifetime history^a^	1.31 (1.07, 1.60)	0.009
Expressed need for childcare^a^	1.24 (1.00, 1.53)	0.050
Expressed need for public housing^a^	1.59 (1.31, 1.95)	< 0.001
**Variables Not Included in the Final Model**		
White racial identification^a^	-	0.409
Pregnancy marijuana use^a^	-	0.178
Past 30-day suicidality^a^	-	0.205
Expressed need for family health care provider^a^	-	0.373
Expressed need for own mental health care^a^	-	0.735
Expressed need for emergency housing^a^	-	0.112

^a^Indicates a dichotomous variable. Values in the table are adjusted odds ratios controlling for all of the other variables (as shown) in the logistic regression model

## Discussion

We examined whether a three-year, personalized, relationship-based intervention can help reduce racial/ethnic disparities in a population of at-risk women. Our results suggest that it can. Among participants who completed the intervention and provided exit data for analysis we found no significant racial/ethnic differences in key outcomes. Given their difficult life circumstances at intake, participants generally were doing remarkably well at exit. Nearly 90% had received SUD treatment. Over three quarters were abstinent from illicit drugs for 30 days or longer at exit and/or had a period of drug and alcohol abstinence of six months or more during the program. Most (70.1%) were regularly using a birth control method. Two-thirds had physical custody of the child that brought them into PCAP and/or were stably housed. Over half (50.4%) had participated in training or education.

To the central question of this study, PCAP achieved comparable outcomes across racial/ethnic groups despite not being explicitly culturally tailored; PCAP does not specify best practices for work with participants based on their cultural groups. Rather, PCAP is individualized based on a participant’s history, circumstances, strengths, and goals. Case managers meet women where they are — both literally and figuratively — and adapt intervention dosage (i.e., their time spent) to participants’ needs. Hispanic women received more direct time with case managers, which may have been related to their having more of their children living with them. Participants of different racial/ethnic groups showed comparable positive outcomes within the context of a supportive relationship with an active, caring, and nonjudgmental case manager, one who was not necessarily of the same race but who connected them to relevant social and community services that were relevant and meaningful to the mother and her family. While racial/ethnic identities vary, most PCAP participants share a common social identity insofar as they are mothers with substance use disorders living in debilitating poverty. Our findings suggest that PCAP may be effective irrespective of a participant’s race/ethnicity by using intervention strategies that help participants overcome obstacles typically associated with this shared social identity; case managers help by eliciting clients’ needs and facilitating getting needs met. Hispanic women, who were significantly more likely to live in rural areas, expressed similar needs for mental health services and alcohol/drug support as other racial/ethnic groups, but they were less likely to have those needs met. Previous analyses of PCAP outcomes have demonstrated similar rural-urban differences [26].

As noted, some participants did not complete the three-year program, for a variety of reasons. We found no racial/ethnic differences in the number of participants that moved out of the service area, went into long-term incarceration, or died (the latter two being unfortunate realities of working with persons with severe SUD). It is likely that some participants for whom the intervention program wasn’t working dropped out, and this could have obscured racial/ethnic differences. About 15% of those enrolled stopped responding to their case manager during the intervention, and another 5% explicitly asked to withdraw. Reasons for disengagement and withdrawal were not reliably recorded, but reflected both positive and negative circumstances, e.g., doing well and not needing PCAP anymore versus relapsing into active addiction and not wanting PCAP anymore. PCAP strongly emphasizes outreach and engagement to participants who disengage, but attrition nonetheless remains a challenge, as might be expected with a three-year intervention.

Of particular concern with regard to disengagement and withdrawal is our finding that, controlling for other factors that predicted attrition, American Indian or Alaska Native participants were less likely than other participants to complete the intervention (Adjusted Odds Ratio = 0.66). (A trend for Black participants not to complete the intervention did not reach significance.) The disproportionate attrition among AI/AN (and perhaps Black participants) could be due to aspects of the program in general, or of the site of their enrollment, or due to the nature of their lives — affected as they may be by structural racism, historical trauma, and personal trauma. We found evidence suggestive of these effects in our examination of maternal history and mental health. AI/AN women reported disproportionate hardships as children, while Black women reported disproportionate psychiatric symptoms. Racism and trauma would certainly be expected to affect — among other things — a woman’s inclination to trust and allow an outsider (even a well-meaning one) into her life. Indeed, AI/AN participants were significantly less likely to complete the program, although, as a group, they received more “other” time from case managers, that is, time spent working with an agency on behalf of the participant, time spent with participant’s family, and time spent corresponding with the participant.

It is possible that AI/AN participants did not remain in PCAP for the full three years because they preferred the supports available within their own tribal communities. We found some evidence of this by examining tribal enrollment information for N = 568 AI/AN participants for whom tribal enrollment status was known: Among those who completed PCAP, 50.8% reported being an enrolled member of a tribe compared to 62.1% among those who attritted (*p* < .05, two-sided test),^1^ suggesting that there may be some association with participants’ tribal membership and their depending on services alternative to PCAP. Qualitative research would be beneficial to examine the perspectives of AI/AN participants enrolled in the program to gain insight into factors affecting their likelihood of completing the program or dropping out. It should be noted that nearly half of the AI/AN participants (*n* = 329, 49%) did complete the program and provide exit data, which we view as a strength of this study.

### Limitations

In interpreting the racial/ethnic differences revealed in our analyses, it is important to keep in mind that our sample is not necessarily representative. Many different factors may have influenced whether an individual was referred to and ultimately participated in PCAP. Ours was not an intent-to-treat analysis; only those women who completed the program were contacted for an exit interview. It is unknown how those who did not compete the program were doing at the three-year follow-up point, nor did we have a control group not receiving PCAP. Future studies should address these limitations. It should also be noted that the present study spans a long time frame during which retail recreational cannabis sales became legal in the state of Washington. While the present analysis did not take this into account, a previous analysis found that cannabis use at program exit increased significantly post cannabis legalization [27]. We did not examine potential effects of time on the outcomes.

### Strengths

Our study has important strengths. Examining data from across the State of Washington collected over a decade provided for a nuanced analysis of racial/ethnic differences among women in a unique case management program for mothers with problematic substance use during pregnancy. The large number of American Indian or Alaska Native women in our sample overall (*n* = 669) enabled insights into the challenges faced by this population and in serving this population. Our findings suggest that the PCAP intervention has comparable benefit across racial/ethnic groups for those who complete it. We have yet to learn whether benefits are observable even among those who do not.

### Implications

Other results of our attrition analysis point to notable considerations for policy makers and others who work with a similar population. Mothers were more likely to remain engaged and complete the program when they had physical custody of their child at intake. Conversely, removing children from mothers’ custody may have a negative impact on their motivation to achieve and maintain recovery from SUDs. Expressing a need for childcare and housing at intake were also associated with completing the program. Needing such assistance may be an important motivator to engaging with case management programs such as PCAP because without such help, meeting childcare and housing needs can be a significant challenge for mothers struggling with poverty and substance use. Having a history of physical abuse was associated with program completion, perhaps because PCAP’s personalized, long-term assistance helped women to address ongoing and complex effects of abuse-related trauma.

## Data Availability

The dataset used in the current study is available from the corresponding author on reasonable request and may be provided with permission from the Washington State Health Care Authority.

## Abbreviations

AI/AN: American Indian/Alaska Native
AOR: Adjusted Odds Ratio
ASI: Addiction Severity Index, 5^th^ Edition
Asian/PI: Asian/Pacific Islander
Black: African American/Black
DCYF: Department of Children, Youth and Families of Washington State
Hispanic: Hispanic/Latino
PCAP: Parent-Child Assistance Program
SUD: Substance use disorder
WSRDAC/M: Washington State Racial Disproportionality Advisory Committee, Modified

## Footnotes

1 These results are similar (at *p* < .05) if the analysis is restricted to only participants in either the Attrition or the Outcome Group (N = 465), i.e., excluding those who finished with no exit interview.
